# A SARS-CoV-2 (COVID-19) biological network to find targets for drug repurposing

**DOI:** 10.1038/s41598-021-88427-w

**Published:** 2021-04-30

**Authors:** Mahnaz Habibi, Golnaz Taheri, Rosa Aghdam

**Affiliations:** 1 Department of Mathematics, Qazvin Branch, Islamic Azad University, Qazvin, Iran; 2 Department of Electrical Engineering and Computer Science, KTH Royal Institute of Technology, Stockholm, Sweden; 3 Science for Life Laboratory, Stockholm, Sweden; 4 School of Biological Sciences, Institute for Research in Fundamental Sciences (IPM), Tehran, Iran

**Keywords:** Computational biology and bioinformatics, Drug discovery, Systems biology

## Abstract

The Coronavirus disease 2019 (COVID-19) caused by the SARS-CoV-2 virus needs a fast recognition of effective drugs to save lives. In the COVID-19 situation, finding targets for drug repurposing can be an effective way to present new fast treatments. We have designed a two-step solution to address this approach. In the first step, we identify essential proteins from virus targets or their associated modules in human cells as possible drug target candidates. For this purpose, we apply two different algorithms to detect some candidate sets of proteins with a minimum size that drive a significant disruption in the COVID-19 related biological networks. We evaluate the resulted candidate proteins sets with three groups of drugs namely Covid-Drug, Clinical-Drug, and All-Drug. The obtained candidate proteins sets approve 16 drugs out of 18 in the Covid-Drug, 273 drugs out of 328 in the Clinical-Drug, and a large number of drugs in the All-Drug. In the second step, we study COVID-19 associated proteins sets and recognize proteins that are essential to disease pathology. This analysis is performed using DAVID to show and compare essential proteins that are contributed between the COVID-19 comorbidities. Our results for shared proteins show significant enrichment for cardiovascular-related, hypertension, diabetes type 2, kidney-related and lung-related diseases.

## Introduction

The global impact of the Coronavirus disease 2019 (COVID-19) pandemic has brought an urgent need for finding treatments to reduce morbidity and mortality. Repurposing existing drugs is an effective and fast way to prepare such treatments by finding a new use for drugs that already have safety and pharmacological profiles^1^. Drug repurposing can be done using different drug development methods. In the COVID-19 situation, drug repurposing can be a fast and cost-effective approach to find novel treatments. Recent studies have increasingly used computational methods to algorithmically predict new drug targets or drug repurposing candidates. Focusing on drug targets that are already approved clinically and evaluating their therapeutic potential for COVID-19 can be one of the fastest solutions. Fehr et al.^2^ revealed that SARS-CoV-2 infects human cells by hijacking the host’s translation mechanism to produce 29 viral proteins. These 29 proteins bind to multiple human proteins to set up the molecular processes that needed for viral duplication and additional host infection. Gorden et al.^3^ used affinity purification mass spectrum method to find interactions between a map from human and SARS-CoV-2 proteins. This study released the 26 proteins from 29 proteins that the SARS-CoV-2 infects in the human body. They also identified 332 human proteins involve in these viral proteins binds. Among these 332 proteins, 67 druggable human proteins with 69 existing drugs identified. The identification of dependencies between host proteins and virus infection can provide significant insights into finding suitable drug targets for developing antivirals medicine against SARS-CoV-2. Saha et al.^4^ described the probable molecular mechanism of Remdesivir as one of the best drug candidates for COVID-19. They showed the effect of Remdesivir with abroad spectrum of anti-viral activity against many viruses, to inhibit the RNA synthesis of SARS-CoV-2. Patel et al.^5^ constructed a tripartite network-based for repurposing the approved drugs to treat COVID-19 patients. Their study showed that the anti-viral properties of resveratrol against SARS-CoV-2 virus could be readily exploited to effectively control the viral load at the early stages of COVID-19 infection. Saha et al.^6^ took a brief look at the development stages of different vital drug candidates, that were being tested as potential vaccines or therapeutics against COVID-19.

Analysis of this large amount of data in the biological process helped us for a better understanding of cellular mechanisms. This kind of data is usually represented in the form of a network. One of the most important biological networks constructed from experimental data is the protein–protein interaction (PPI) network^7–9^. The graph-based analysis of PPI networks with respects to different human diseases has resulted in the identification of appropriate drug target proteins^10, 11^. The previous studies demonstrated that the relationship between essential proteins in the biological network along with some graph-based properties^12, 13^. Most of the essential proteins have a high degree in a network^13^. Another important graph-based property in the network is the betweenness centrality value^12^. The value of betweenness for each node in the network represents the total number of the shortest pathways that pass through this node in the network.

Recent studies showed that removing the essential proteins disrupt the vital biological processes in the cell and may be lethal to an organism^14^. Some computation methods designed informative networks from biological processes data to identify essential proteins with important biological properties. For this purpose two algorithms^15, 16^ are applied. These algorithms detect the minimum number of proteins from biological networks that lead to a major disruption in the network.

In the first part of this work, we construct a biological network as a weighted simple graph related to virus targets or their associated biological processes. Then, we use two effective algorithms^15, 16^ to find the minimum number of proteins from biological networks that lead to a major disruption in the network. The selection methods for essential nodes in the first and second algorithms are based on the betweenness value for each node in a weighted graph and the spectral partitioning in the Laplacian graph, respectively. We evaluate our candidate sets as essential proteins related to COVID-19 with three groups of drugs namely Covid-Drug, Clinical-Drug, and All-Drug. We show that 16 drugs out of 18 in the Covid-Drug and 273 drugs out of 328 in the Clinical-Drug are approved by our method. Also, our candidate sets approve a large number of drugs in All-Drug.

In the second part of this work, we identify proteins in our candidate sets that are associated with some underlying diseases related to COVID-19. At the end, we find 93 proteins as a final set of essential proteins related to disease pathology. It can be concluded that our candidate proteins are targeted by a large number of COVID-19 drugs. We also show some significant signaling and disease pathways.

## Results

To identify the best proteins set as a drug target, we propose a two-step method. In the first step, we try to detect essential proteins from SARS-CoV-2 virus targets or their associated modules in human cells. Then, in the second step, we try to find essential proteins that are related to comorbid disease pathologies. To construct our sets, we consider 1374 Informative Biological Process (IBP) Gene Ontology (GO) terms related to 332 human proteins identified in^3^ as high-confidence virus human protein interactions. In order to prioritize proteins that can be essential proteins sets related to COVID-19, *T*, $Cut_1$, $Cut_2$, $C_1$, $C_2$, $T_1$, $T_2$ and $Cut_{75}$, $Level_1$, $S_1$, $S_2$, $E_1$ and $E_2$, sets are defined as follows:

- *T*: The set of 332 proteins reported as possible targets of the SARS-CoV-2 virus^3^.
- $Cut_1$: The minimum cut set resulted from Algorithm 1^16^.
- $Cut_2$: The minimum cut set resulted from Algorithm 2^15^.
- $C_1$: The elements of $Cut_1$ that physically interacted with the SARS-CoV-2 virus (intersection of $Cut_1$ and *T*).
- $C_2$: The elements of $Cut_2$ that physically interacted with the SARS-CoV-2 virus (intersection of $Cut_2$ and *T*).
- $T_1$: Intersection of $C_1$ and $C_2$.
- $T_2$: Union of $C_1$ and $C_2$.
- $Cut_{75}$: From all of the proteins in the $Cut_1$ or $Cut_2$ that have the highest degree and the highest number of disruption, 75 most important proteins are selected.
- $Level_1$: The neighbors of *T* set.
- $S_1$: Intersection of $Cut_1$ and $Cut_2$.
- $S_2$: Union of $Cut_1$ and $Cut_2$.
- $E_1$: Set of essential proteins associated with COVID-19 that are placed in $Cut_1$.
- $E_2$: Set of essential proteins associated with COVID-19 that are placed in $Cut_2$.

**Table 1:** Description of the defined sets.

Set	Description
*T*	The set of 332 proteins reported as possible targets of the SARS-CoV-2 virus^3^
$Cut_1$	The minimum cut set resulted from Algorithm 1^16^
$Cut_2$	The minimum cut set resulted from Algorithm 2^15^
$C_1$	The elements of $Cut_1$ that physically interacted with the SARS-CoV-2 virus (intersection of $Cut_1$ and *T*)
$C_2$	The elements of $Cut_2$ that physically interacted with the SARS-CoV-2 virus (intersection of $Cut_2$ and *T*)
$T_1$	Intersection of $C_1$ and $C_2$
$T_2$	Union of $C_1$ and $C_2$
$Cut_{75}$	From all of the proteins in the $Cut_1$ or $Cut_2$ that have the highest degree and the highest number of disruption, 75 most important proteins are selected
$Level_1$	The neighbors of set *T*
$S_1$	Intersection of $Cut_1$ and $Cut_2$
$S_2$	Union of $Cut_1$ and $Cut_2$
$E_1$	Set of essential proteins associated with COVID-19 that are placed in $Cut_1$
$E_2$	Set of essential proteins associated with COVID-19 that are placed in $Cut_2$

Best results are indicated in bold.

**Figure 1:**
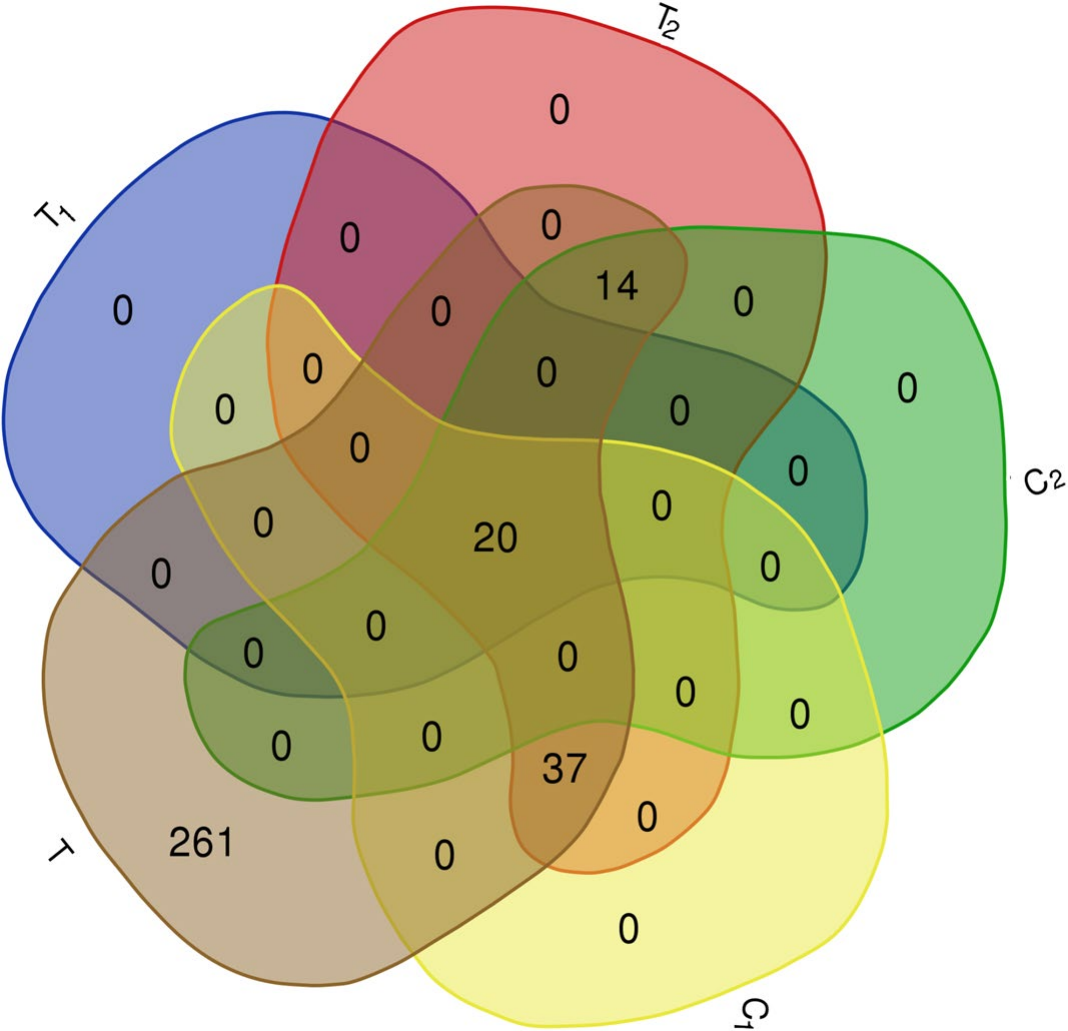
The Venn diagram of $T_1$, $T_2$, $C_1$, $C_2$ and *T* sets. The complete description of sets is presented in Table 1.

**Table 2:** The summary of statistics of the proposed sets.

	$T_1$	$C_1$	$C_2$	$T_2$	$Cut_{75}$	*T*	$S_1$	$Cut_1$	$Cut_2$	$S_2$	$Level_1$
No. proteins	20	57	34	71	75	332	1115	2017	2100	3002	7845
Mean of degrees	122.45	95.41	96.44	88.28	**406**.**48**	59.65	78.09	69.91	59.12	59.32	59.71
IBP GO terms	439	857	557	975	1384	1870	10,378	14,642	13,726	17,990	**18,052**
Unique IBP GO terms	400	737	493	821	643	**1374**	1120	1279	1197	1306	1364
Percentage of Unique IBP GO terms	**0**.**91**	0.85	0.88	0.84	0.46	0.73	0.11	0.08	0.08	0.07	0.07
Average Unique IBP GO terms	**20**	12.92	14.5	11.56	8.57	4.13	1.00	0.63	0.57	0.43	0.17

The first row shows the size of $T_1$, $C_1$, $C_2$, $T_2$, $Cut_{75}$, *T*, $S_1$, $Cut_1$, $Cut_2$, $S_2$, $Level_1$ sets. The second row shows the mean of degrees of mentioned sets. The number of 1374 IBP GO terms overlapped with the subsets and their number of unique IBP GO terms are collected in the third and fourth rows, respectively. The ratio of the unique IBP GO terms to total IBP GO terms and the ratio of the number of unique IBP GO terms to the size of the selected set are represented in the fifth and sixth rows, respectively. The complete description of sets is presented in Table 1. Best results are indicated in bold.

The complete description of *T*, $Cut_1$, $Cut_2$, $C_1$, $C_2$, $T_1$, $T_2$, $Cut_{75}$, $Level_1$, $S_1$, $S_2$, $E_1$ and $E_2$ sets is presented in Table 1.

**Figure 2:**
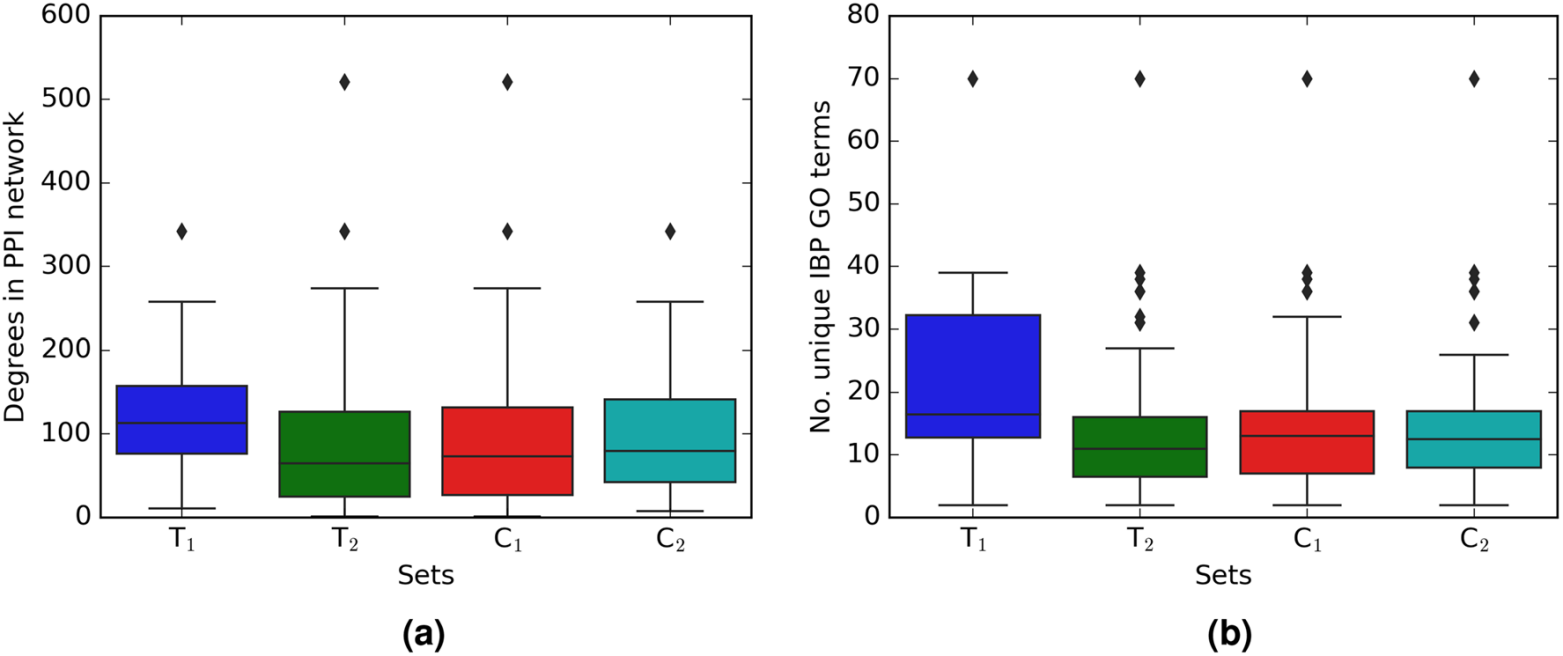
(**a**) Boxplot of the degrees in protein–protein interaction (PPI) network for $T_1$, $T_2$, $C_1$, and $C_2$ sets; (**b**) Boxplot of the number of unique IBP GO terms resulted from each of the four sets. The complete description of sets is presented in Table 1.

### Evaluation of our proposed essential proteins subsets with respect to the number of disruption

The Venn diagram for $T_1$, $T_2$, $C_1$, $C_2$ and *T* sets is illustrated in Fig. 1 (http://bioinformatics.psb.ugent.be/webtools/Venn/). This figure shows that from 332 proteins in *T* set, only 71 proteins are selected with mentioned algorithms ($T_2$). From these 71 proteins, 20 proteins are selected in both of mentioned algorithms ($T_1$) (see “Methods” section), 37 proteins are selected uniquely in Algorithm 1 ($C_1 \backslash T_1$) and 14 proteins are selected uniquely in Algorithm 2 ( $C_2 \backslash T_1$). The results of mentioned sets are summarized in Table 2. The number of proteins in $T_1$, $C_1$, $C_2$, $T_2$, $Cut_{75}$, *T*, $S_1$, $Cut_1$, $Cut_2$, $S_2$ and $Level_1$ sets are reported in the first row. The second row shows the mean of degrees of the mentioned sets. The third row shows the number of 1374 IBP GO terms overlapped with our sets and their number of unique IBP GO terms are collected in the fourth row (see “Dataset” in the “Methods” section)^17^. According to Table 2, for example, the number of unique IBP GO terms for $T_1$ with a size of 20 is equal to 400, and similarly for $T_2$ with a size of 71 is equal to 821. The ratio of the number of unique IBP GO terms to the total IBP GO terms indicates that the degree of uniqueness of the introduced proteins sets are represented in the fifth row. The ratio of the number of unique IBP GO terms to the size of the selected set represents on average, each protein causes how many unique disruption. $T_1$ has the highest percentage of unique IBP GO terms and also, the ratio of the number of disruption on average in comparison with other sets. The ratio of the number of unique IBP GO terms to the size of the selected set is shown in the sixth row of the table. Compared to the $Cut_{75}$ set, we can conclude that the proposed algorithms^15, 16^ are not just based on high degrees and high disruption, also some valuable properties are used in them to select the important proteins.

Figure 2 shows the boxplots of the degrees in PPI network (part (a)) and the unique number of IBP GO terms (part (b)) that are resulted from $T_1$, $T_2$, $C_1$, and $C_2$, respectively. As shown in Fig. 2, the degrees of selected sets are similarly distributed, and the majority of the number of disruption is located between 0 and 40 with a maximum value of 70. The median of the number of unique disruption related to $T_1$ is bigger than the other sets.

**Figure 3:**
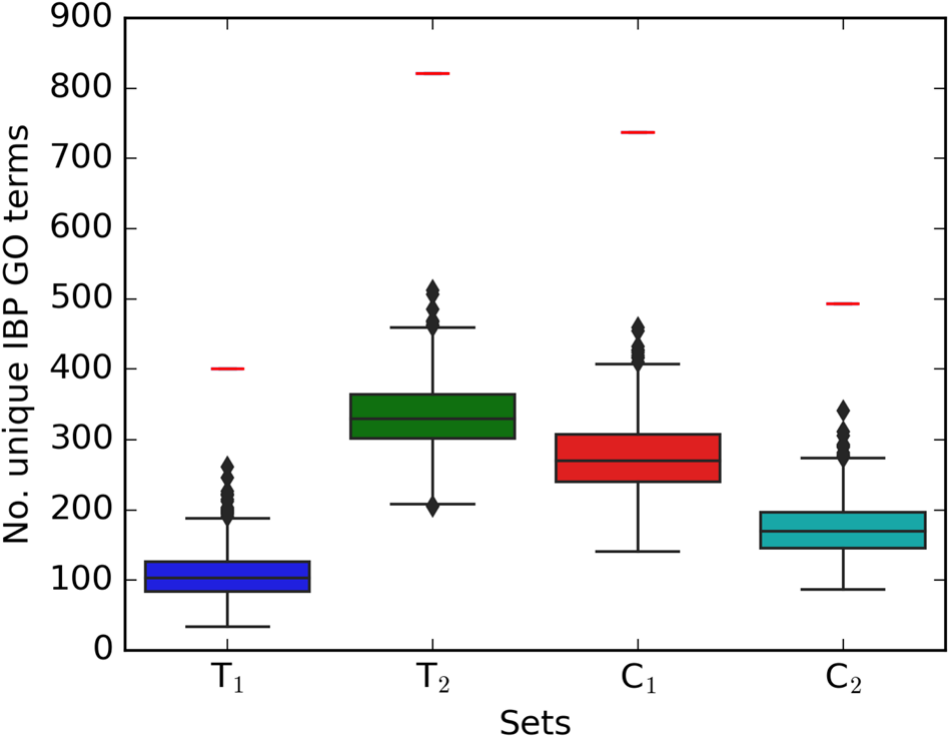
Boxplot of number of unique IBP GO terms resulted from 1000 randomly selected sets of sizes 20, 71, 57, and 34, respectively. Red small lines in the figure are the number of unique IBP GO terms for $T_1$, $T_2$, $C_1$ and $C_2$ sets. The complete description of sets is presented in Table 1.

**Table 3:** The summary of drug targets and related drugs for Covid-Drug group.

	$T_1$	$C_1$	$C_2$	$T_2$	*T*	$S_1$	$Cut_1$	$Cut_2$	$S_2$	$Level_1$
No. proteins	20	57	34	71	332	1115	2017	2100	3002	7845
No. proteins targets	0	1	0	1	1	15	22	20	27	**34**
No. drugs	0	2	0	2	2	14	15	15	**16**	14
Ratio of the proteins targets	0	**0**.**017**	0	0.014	0.003	0.013	0.011	0.009	0.008	0.004
Ratio of drugs	0	**0**.**035**	0	0.028	0.006	0.0125	0.007	0.007	0.005	0.001

The first row shows the size of $T_1$, $C_1$, $C_2$, $T_2$, *T*, $S_1$, $Cut_1$, $Cut_2$, $S_2$, $Level_1$ sets. The number of proteins targets and related drugs in each set for Covid-Drug group are reported in second and third rows, respectively. The fourth and fifth rows show the ratio of the number of proteins that targeted and their related drugs in each set for Covid-Drug group to the size of sets, respectively. The complete description of sets is presented in Table 1. Best results are indicated in bold.

**Figure 4:**
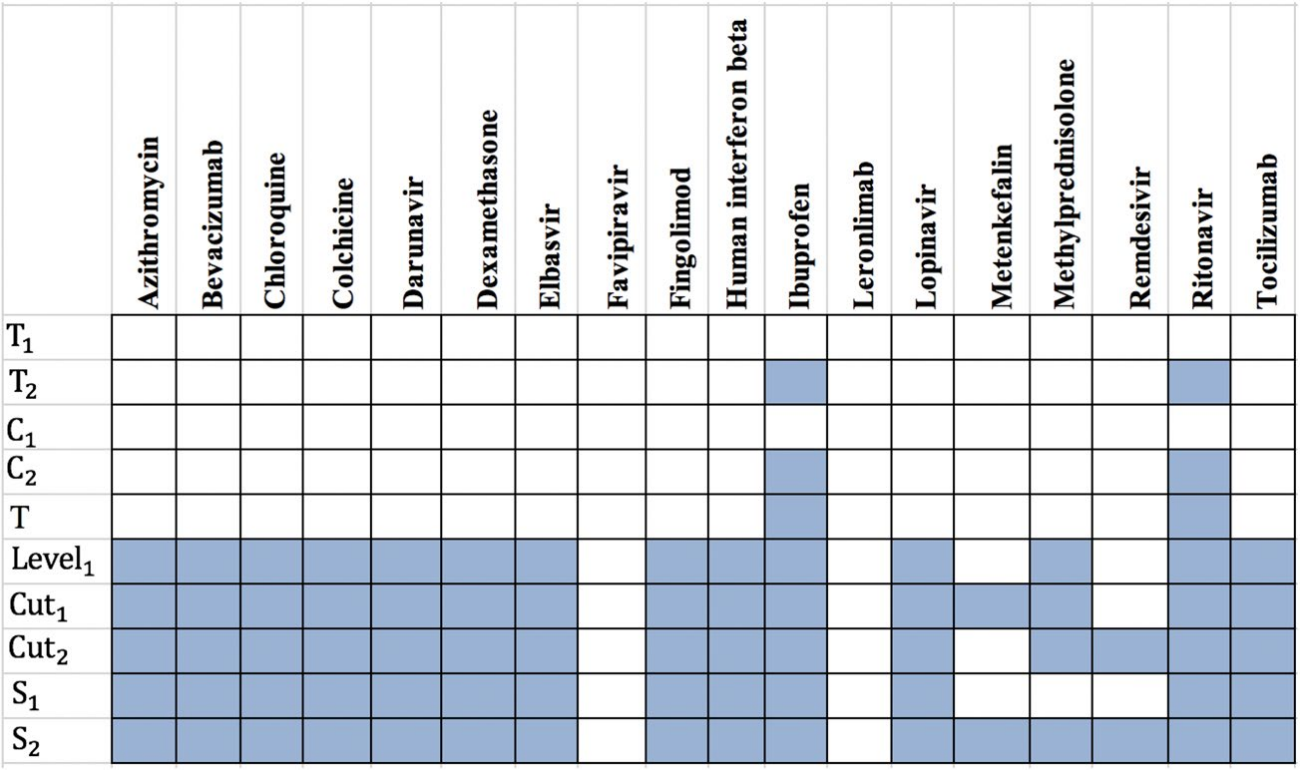
The presence (blue color) or absence (white color) of the overlap of proteins targets of $T_1$, $T_2$, $C_1$, $C_2$ *T*, $Level_1$, $Cut_1$, $Cut_2$, $S_1$ and $S_2$ sets with the targets of Covid-Drug. The complete description of sets is presented in Table 1.

In order to evaluate the performance of the mentioned algorithms, we compare our selected subsets from *T* ($T_1$, $T_2$, $C_1$ and $C_2$) with randomly generated subsets. For each of the proposed sets ($T_1$, $T_2$, $C_1$ and $C_2$) of size *n*, $10^3$ proteins sets are generated as possible targets of SARS-CoV-2 virus from *T* with size *n*. Suppose that $N_i$ for $i = 1,\ldots , 10^3$ are the number of GO terms (from 1374) that disrupted with randomly generated set and *N* is the number of unique IBP GO terms resulted from our sets. Let $X = \{i | N_i > N \}$ for $i = 1,\ldots , 10^3$ where X denotes the number of random results that performed better than the output of the two mentioned algorithms. The null hypothesis, $H_0$, is that our selected proteins set of size *n* is not important. The alternative hypothesis, $H_1$, is that our selected proteins set of size *n* is indeed important. We use Exceeding Value as $EV=\dfrac{|X|}{1000},$ where |*X*| denotes the size of *X*^18^. If $EV<\alpha$ then, we reject $H_0$ ($\alpha$ is a threshold value that we consider to be 0.05). The values of EV for all selected proteins sets are equal to zero (This value causes extremely significant results). We can conclude that the results of mentioned algorithms show a better performance than all of these random selections. Figure 3 illustres the boxplots of the number of disruption resulted from 1000 randomly selected sets of sizes 20, 71, 57, and 34, respectively. The small red lines above each boxplot in this figure shows the number of unique IBP GO terms related to $T_1$, $T_2$, $C_1$, and $C_2$ which are equal to 400, 821, 737, and 493, respectively (see Table 2). As shown in this figure, the results of random selections are significantly less than our results. It means that there is no random set that performs better than our selected set. It can be concluded that the results are significantly far from random and by choosing the appropriate sets, a significant amount of disruption happens.

**Figure 5:**
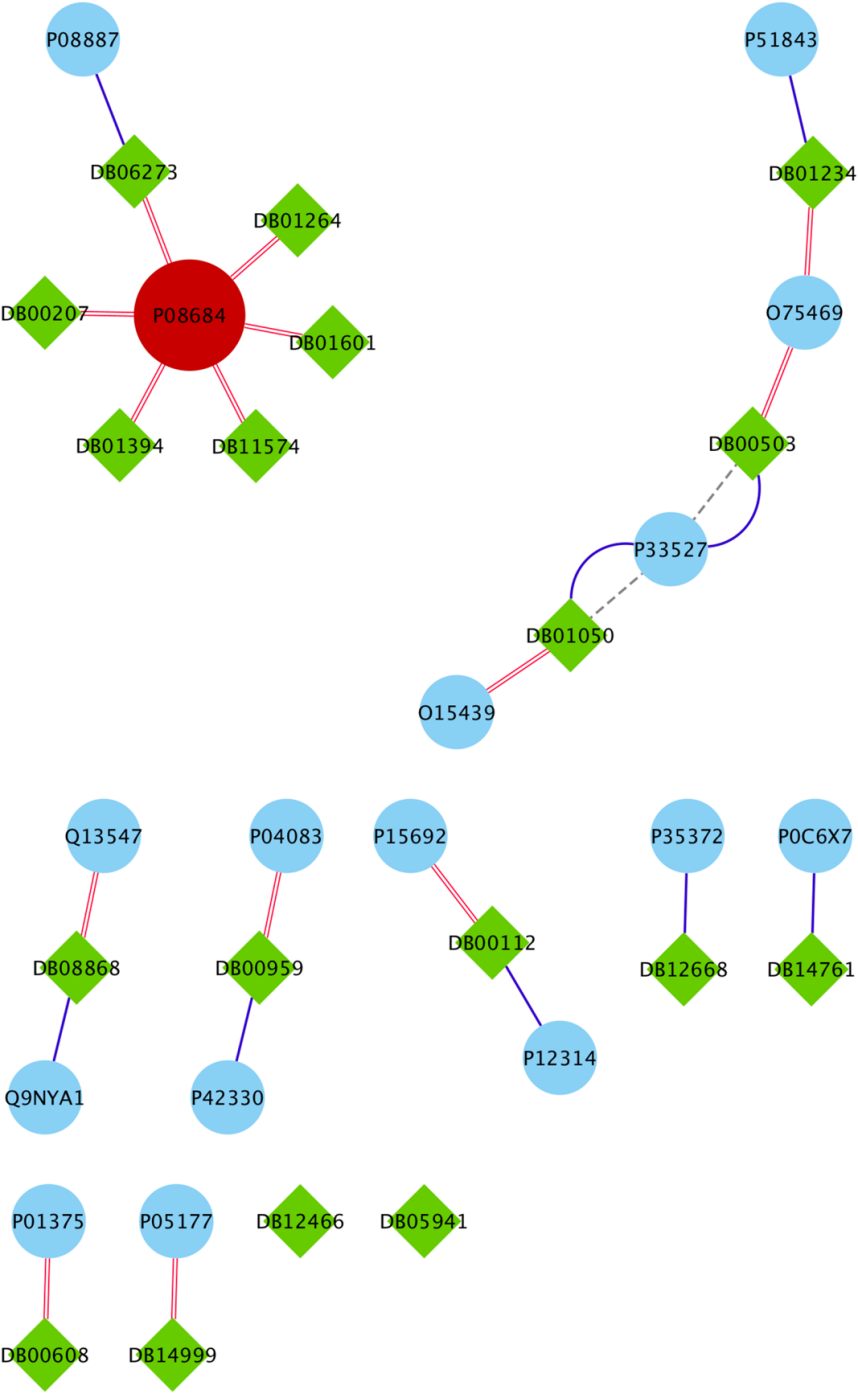
The drug targets in Covid-Drug group for $T_2$, *L* ($Level_1 \backslash T_2$), and *C* ( other proteins of $S_2$ except $S_2 \backslash L$ ) sets. The red color node indicates the protein that is the target of a large number of drugs. The red edges, blue edges and black dotted edges are related to *L*, *C* and $T_2$ sets, respectively. The complete description of sets is presented in Table 1.

**Table 4:** The summary of drug targets and related drugs for Clinical-Drug group.

	$T_1$	$C_1$	$C_2$	$T_2$	*T*	$S_1$	$Cut_1$	$Cut_2$	$S_2$	$Level_1$
No. proteins	20	57	34	71	332	1115	2017	2100	3002	7845
No. proteins targets	1	4	2	5	15	154	218	217	281	**398**
No. drugs	2	17	4	19	30	225	246	260	273	**284**
Ratio of the proteins targets	0.05	0.070	0.058	0.070	0.045	0.138	0.108	0.103	**0**.**093**	0.051
Ratio of drugs	0.1	**0**.**298**	0.117	0.267	0.090	0.201	0.1215	0.121	0.091	0.036

The first row shows the size of $T_1$, $C_1$, $C_2$, $T_2$, *T*, $S_1$, $Cut_1$, $Cut_2$, $S_2$, $Level_1$ sets. The number proteins targets and related drugs in each set for Clinical-Drug group are reported in second and third rows, respectively. The fourth and fifth rows show the ratio of the number of proteins that targeted and their related drugs in each set for Clinical-Drug group to the size of sets, respectively. The complete description of sets is presented in Table 1.

**Figure 6:**
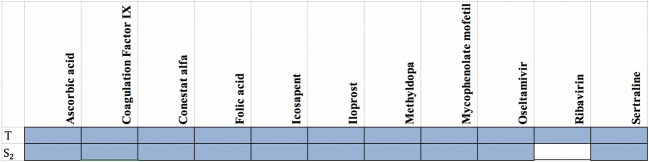
The presence (blue color) or absence (white color) of overlap of approved drugs in *T* and $S_2$ sets that are not approved with any of the proteins in $T_2$ set. The complete description of sets is presented in Table 1. Best results are indicated in bold

### Evaluation of our proposed essential proteins subsets with respect to the related drugs

To justify our proposed essential proteins, we evaluate 37 experimental unapproved drugs for COVID-19 that are reported in DrugBank^19^. From these 37 drugs, 19 drugs have no targets information and only 18 drugs have the drug target information from our PPI network that denoted as Covid-Drug. These 18 drugs have 78 proteins targets in our PPI network. It is worth mentioning that just one of these proteins is from *T* set (**P33527** protein). We find that from these 18 drugs in Covid-Drug, only two of them have this target in 332 proteins including **Ritonavir** and **Ibuprofen**. Both of these drug targets are approved with mentioned algorithms. In other words, this target is determined with $T_2$ to be one of the significant targets in mentioned algorithm. We also find that all drugs in this group except **Favipiravir** and **Leronlimab** have targeted at least one proteins in our cut sets, while the $Level_1$ set with 7845 proteins are targeted with 14 drugs in Covid-Drug. The details of some statistical information of our candidate essential proteins subsets for Covid-Drug group are reported in Table 3. In this table, the first row indicates the size of $T_1$, $C_1$, $C_2$, $T_2$, *T*, $S_1$, $Cut_1$, $Cut_2$, $S_2$ and $Level_1$ sets, respectively. The number of proteins targets and related drugs for Covid-Drug group are reported in the second and third rows, respectively. The fourth and fifth rows show the ratio of the number of proteins that are targeted and their related drugs for Covid-Drug group to the size of sets, respectively. The results of presence (blue color) or absence (white color) of overlaps in proteins targets of $T_1$, $T_2$, $C_1$, $C_2$, *T*, $Level_1$, $Cut_1$, $Cut_2$, $S_1$ and $S_2$ sets with the targets of Covid-Drug are shown in the Fig. 4. For a better evaluation of mentioned algorithms, the drug targets and related drugs in Covid-Drug group are illustrated in Fig. 5 . In this figure, the green diamond nodes indicate the drugs and the blue circle nodes show the targets associated with these drugs. The protein that is the target of a large number of drugs is shown in red color. Figure 5 shows the distribution of drug targets in mentioned algorithm for three separate subsets of $S_2$. The first subset contains a protein that targeted by the virus ($T_2$), the second subset contains some proteins that are located in $Level_1 \backslash T_2$ (shown as *L*) and other proteins of $S_2$ are located in the third subset (*C*). It is noticeable that the **P08684** protein is shown in red color, is one of the targets for most drugs in the Covid-Drug group. The red, blue, and black dotted edges are related to *L*, *C*, and $T_2$ sets, respectively.

The second group of drugs contains 449 drugs as clinical trials for COVID-19. From these 449 drugs, 328 drugs have targets in the PPI network denoted as Clinical-Drug. These 328 drugs can target 888 proteins in a cell. From these 888 proteins, 281 proteins are approved with mentioned algorithms. The details of some statistical information about Clinical-Drug are reported in Table 4. In this table, the first row indicates the size of $T_1$, $C_1$, $C_2$, $T_2$, *T*, $S_1$, $Cut_1$, $Cut_2$, $S_2$ and $Level_1$ sets, respectively. The number of proteins targets and related drugs for Clincal-Drug group are reported in the second and third rows, respectively. The fourth and fifth rows show the ratio of the number of proteins that targeted and their related drugs for Clincal-Drug group to the size of sets, respectively. As seen in Table 4, from these 888 proteins, 15 of them are located in *T* set. From the 328 drugs in Clinical-Drug group, 30 drugs have these 15 targets. From these 15 proteins, five of them are approved with mentioned algorithms. On the other hand,19 drugs of this group are approved with the already-mentioned five proteins of $T_2$. From above 30 drugs, 11 ( = 30 − 19) drugs can target proteins in *T* set, these drugs are not approved with the proposed sets, ($T_1$, $T_2$, $C_1$ and $C_2$), which are subsets of *T*. The size of our proposed sets is much smaller than *T* set, it is worth mentioning that despite the small size, they are able to determine important drug targets in the COVID-19. Our results show that 10 out of 11 drugs have been targeted with $S_2$ set (see Fig. 6).

**Figure 7:**
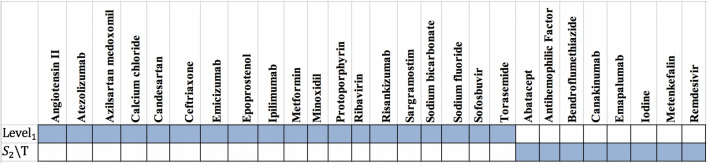
The presence (blue color) or absence (white color) of different approved drugs in $Level_1$ and $S_2 \backslash T$ sets that are not approved with any of the proteins in $S_2$ set.The complete description of sets is presented in Table 1.

**Figure 8:**
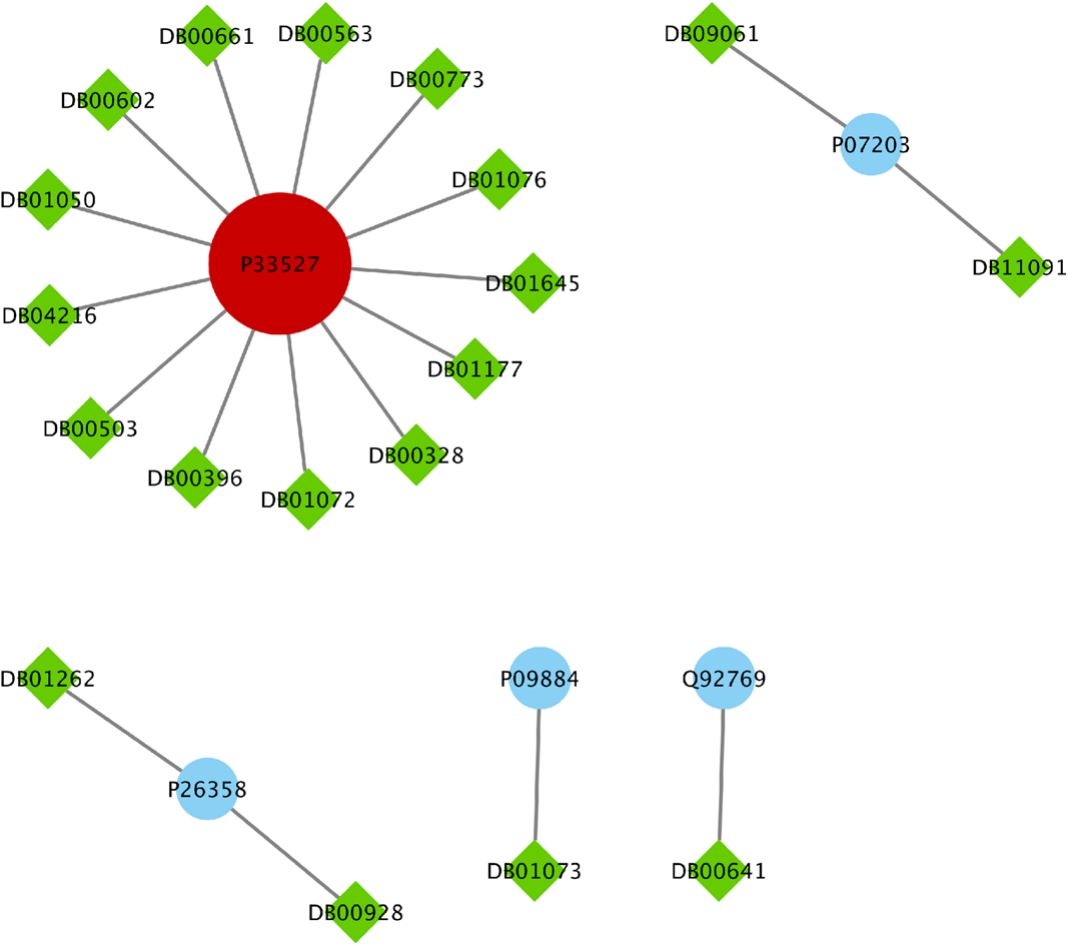
The drug targets in Clinical-Drug group that are located in $T_2$ sets.

**Figure 9:**
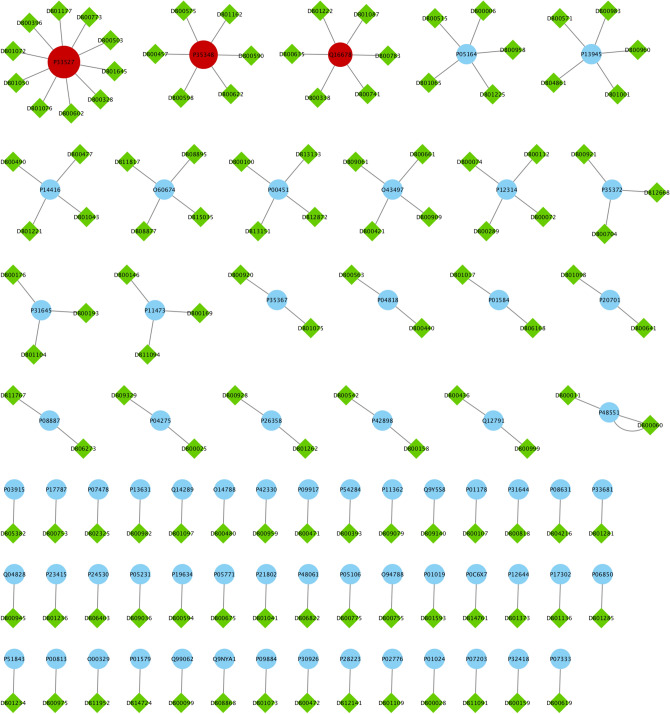
The drug targets in Clinical-Drug group that are located in *L* set.

**Figure 10:**
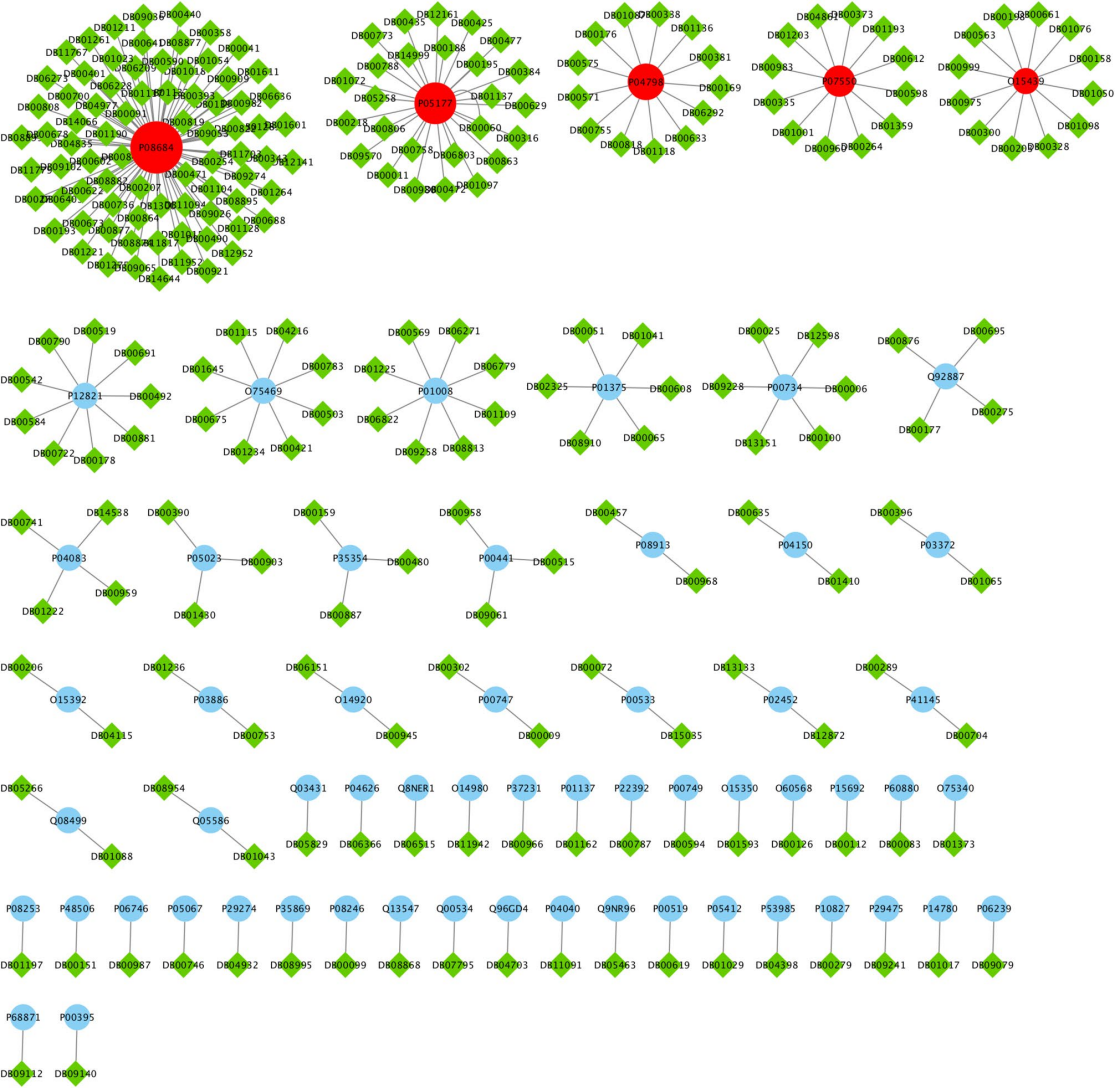
The drug targets in Clinical-Drug group that are located in *C* set.

We also find that from 7,845 proteins in $Level_1$, 398 proteins are targeted by 284 drugs from Clinical-Drug group. It is noticeable that 273 drugs from these 328 drugs are approved with mentioned algorithms. Figure 7 shows that from these 328 Clinical-Drugs, 19 drugs have targets in the $Level_1$ set but are not approved with any of the proteins in $S_2$ set. On the other hand, there are eight other drugs that can target proteins in $S_2$ set but are not approved with any of the proteins in $Level_1$ set. Despite the fact that the size of the recommended sets is much smaller than $Level_1$ set, but the target of these drugs is neither belongs to *T* set nor $Level_1$ set. From these eight drugs, two of them (**Metenkefalin** and **Remdesivir**) are related to a specific drug (Covid-Drug) that is widely used for COVID-19 (see Fig. 4). The drug targets and related drugs in Clinical-Drug group are illustrated in Fig. 8, 9 and 10 . In these figures, the green diamond nodes indicate that the drugs and the blue circle nodes show the targets associated with these drugs. Figure 8 shows the drug targets in Clinical-Drug group that are located in $T_2$ set. The **P33527** protein, shown in red color, is one of the targets of most drugs in the Clinical-Drug group. Figure 9 shows the drug targets in Clinical-Drug group that are located in *L* set. The **P33527**, **P35348** and **Q16678** proteins shown in red color, are the targets of most drugs in the Clinical-Drug. The drug targets in Clinical-Drug group that are located in *C* set are reported in Fig. 10. The **P08684**, **P05177**, **P04798**, **P07550**, and **Q15439** proteins shown in red color, are the targets of most drugs in the Clinical-Drug group.

**Table 5:** The summary of drug targets and related drugs for All-Drug group.

	$T_1$	$C_1$	$C_2$	$T_2$	*T*	$S_1$	$Cut_1$	$Cut_2$	$S_2$	$Level_1$
No. proteins	20	57	34	71	332	1115	2017	2100	3002	7845
No. proteins targets	9	19	15	25	64	356	581	539	764	**1393**
No. drugs	76	166	87	177	274	2831	3345	3297	3754	**4454**
Ratio of the proteins targets	** 0.45**	0.333	0.441	0.352	0.192	0.319	0.288	0.256	0.2545	0.177
Ratio of drugs	**3**.**8**	2.912	2.558	2.492	0.825	2.539	1.658	1.57	1.250	0.567

The first row shows the size of $T_1$, $C_1$, $C_2$, $T_2$, *T*, $S_1$, $Cut_1$, $Cut_2$, $S_2$, $Level_1$ sets. The number proteins targets and related drugs in each set for All-drug group are reported in second and third rows, respectively. The fourth and fifth rows show the ratio of the number of proteins that targeted and their related drugs in each set for All-drug group to the size of sets, respectively. The complete description of sets is presented in Table 1.

**Table 6:** Essential proteins associated with COVID-19 pathology.

	Proteins
$E_1$	O00206, O14543, O60603, P00533, P00734, P01019, P01130, P01133, P01137, P01344
	P01374, P01375, P01579, P01584, P01889, P02647, P02649, P02751, P02778, P03372
	P04114, P04637, P05019, P05089, P05112, P05164, P05231, P05362, P06858, P08571
	P08684, P09211, P09601, P10145, P10415, P10635, P11021, P11473, P13498, P13500
	P13501, P14210, P14780, P15692, P16035, P17813, P19838, P21549, P28482, P29279
	P29459, P29474, P31645, P31749, P35222, P35354, P38936, P40763, P40933, P41597
	P42336, P42345, P42898, P48023, P48061, P60568, P78423, P78527, P81172, Q04721
	Q14116, Q15848, Q16236, Q30201, Q99958, Q9NR96
$E_2$	O00206, O14543, O14763, O60603, P00533, P00734, P01019, P01033, P01130, P01133
	P01137, P01344, P01375, P01579, P01583, P01584, P01889, P01891, P01892, P01911
	P01912, P02647, P02649, P02778, P03372, P03989, P04114, P04229, P04439, P04637
	P05019, P05089, P05106, P05112, P05164, P05231, P05362, P05534, P06858, P08253
	P08684, P09211, P09601, P10415, P10635, P11226, P13498, P13500, P13501, P14210
	P14780, P15692, P16035, P17813, P19438, P19838, P25445, P29279, P29474, P31749
	P35222, P35354, P38936, P40763, P42336, P42345, P48023, P48061, P60568, P78423
	P78527, P81172, Q14116, Q15848, Q16236, Q30201, Q99958, Q9NR96, Q9Y2R2

We also study the number of targets in all drugs reported in UniProt as human drugs that denoted as All-Drug. The summary of drug targets and related drugs are presented in Table 5. In this table, the first row indicates the size of $T_1$, $C_1$, $C_2$, $T_2$, *T*, $S_1$, $Cut_1$, $Cut_2$, $S_2$ and $Level_1$ sets, respectively. The number of proteins targets and related drugs for All-Drug group are reported in the second and third rows, respectively. The fourth and fifth rows show the ratio of the number of proteins that targeted and their related drugs for All-Drug group to the size of sets, respectively. It can be concluded that the proposed candidate proteins sets approve a large number of drugs in All-Drug.

**Figure 11:**
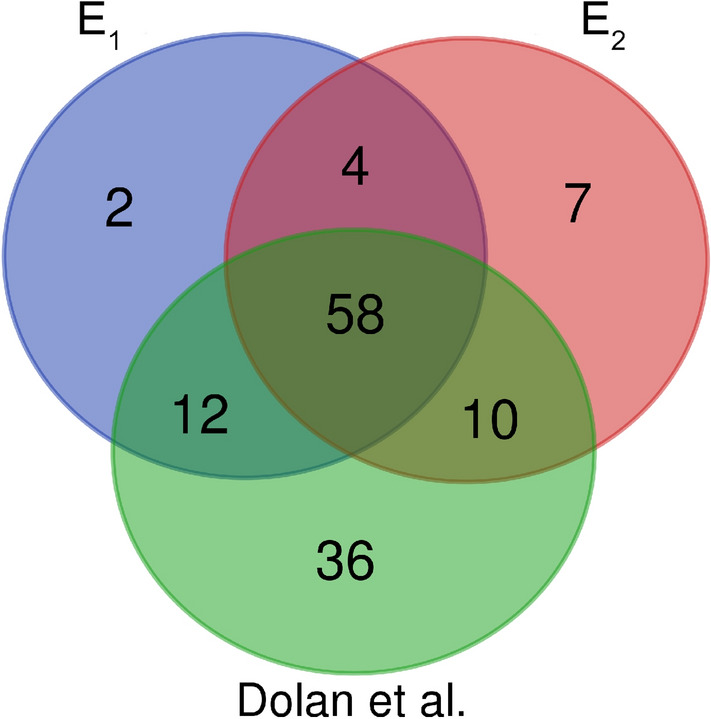
Essential proteins in $E_1$, $E_2$ sets and set of proteins proposed by Dolan et al.^20^ .

**Table 7:** Some of the significantly enriched pathways that are related to COVID-19 essential proteins ($E_1$).

Term	No. gene	P-value	FDR
**Annotation cluster 1 (Enrichment score: 9.739)**			
hsa05142:Chagas disease (American trypanosomiasis)	18	1.15E−16	1.23E−14
hsa05144:Malaria	14	7.52E−16	2.68E−14
hsa05323:Rheumatoid arthritis	15	1.17E−13	2.09E−12
hsa05164:Influenza A	18	7.98E−13	1.22E−11
hsa05321:Inflammatory bowel disease (IBD)	13	1.00E−12	1.34E−11
hsa04620:Toll-like receptor signaling pathway	15	1.68E−12	1.99E−11
hsa05146:Amoebiasis	14	2.99E−11	2.28E−10
hsa05140:Leishmaniasis	12	8.49E−11	5.68E−10
hsa05152:Tuberculosis	16	1.67E−10	9.90E−10
hsa05134:Legionellosis	10	2.55E−09	1.30E−08
hsa05145:Toxoplasmosis	12	1.04E−08	4.82E−08
hsa04064:NF-kappa B signaling pathway	11	1.32E−08	5.89E−08
hsa04621:NOD-like receptor signaling pathway	9	7.35E−08	3.03E−07
hsa05133:Pertussis	9	7.49E−07	2.59E−06
hsa05132:Salmonella infection	9	1.64E−06	4.88E−06
h_nthiPathway:NFkB activation by Nontypeable Hemophilus influenzae	5	0.008042	0.129753
**Annotation cluster 2 (Enrichment score: 4.639)**			
hsa04621:NOD-like receptor signaling pathway	9	7.35E−08	3.03E−07
hsa05020:Prion diseases	5	3.62E−04	6.06E−04
hsa04623:Cytosolic DNA-sensing pathway	6	4.52E−04	7.22E−04
**Annotation cluster 3 (Enrichment score: 3.278)**			
hsa04060:Cytokine–cytokine receptor interaction	19	1.76E−11	1.57E−10
h_cytokinePathway:Cytokine Network	10	1.25E−08	2.12E−06
hsa04940:Type I diabetes mellitus	8	1.69E−07	6.47E−07
h_inflamPathway:Cytokines and Inflammatory response	10	2.09E−07	1.77E−05
hsa05332:Graft-versus-host disease	7	7.97E−07	2.66E−06
hsa05330:Allograft rejection	7	1.62E−06	4.88E−06
hsa04630:Jak-STAT signaling pathway	10	1.40E−05	3.55E−05
22.Cytokine-chemokine_CNS	7	3.54E−05	0.001257
hsa04672:Intestinal immune network for IgA production	6	1.04E−04	2.18E−04

**Table 8:** Some of the significantly enriched pathways that are related to COVID-19 essential proteins ($E_2$).

Term	No. gene	P-value	FDR
**Annotation cluster 1 (Enrichment score: 9.739)**			
hsa05142:Chagas disease (American trypanosomiasis)	18	1.15E−16	1.23E−14
hsa05144:Malaria	14	7.52E−16	2.68E−14
hsa05323:Rheumatoid arthritis	15	1.17E−13	2.09E−12
hsa05164:Influenza A	18	7.98E−13	1.22E−11
hsa05321:Inflammatory bowel disease (IBD)	13	1.00E−12	1.34E−11
hsa04620:Toll-like receptor signaling pathway	15	1.68E−12	1.99E−11
hsa05146:Amoebiasis	14	2.99E−11	2.28E−10
hsa05140:Leishmaniasis	12	8.49E−11	5.68E−10
hsa05152:Tuberculosis	16	1.67E−10	9.90E−10
hsa05134:Legionellosis	10	2.55E−09	1.30E−08
hsa05145:Toxoplasmosis	12	1.04E−08	4.82E−08
hsa04064:NF-kappa B signaling pathway	11	1.32E−08	5.89E−08
hsa04621:NOD-like receptor signaling pathway	9	7.35E−08	3.03E−07
hsa05133:Pertussis	9	7.49E−07	2.59E−06
hsa05132:Salmonella infection	9	1.64E−06	4.88E−06
h_nthiPathway:NFkB activation by Nontypeable hemophilus influenzae	5	0.008042	0.129753
**Annotation cluster 2 (Enrichment score: 4.639)**			
hsa04621:NOD-like receptor signaling pathway	9	7.35E−08	3.03E−07
hsa05020:Prion diseases	5	3.62E−04	6.06E−04
hsa04623:Cytosolic DNA-sensing pathway	6	4.52E−04	7.22E−04
**Annotation cluster 3 (Enrichment score: 3.278)**			
hsa04060:Cytokine–cytokine receptor interaction	19	1.76E−11	1.57E−10
h_cytokinePathway:Cytokine Network	10	1.25E−08	2.12E−06
hsa04940:Type I diabetes mellitus	8	1.69E−07	6.47E−07
h_inflamPathway:Cytokines and inflammatory response	10	2.09E−07	1.77E−05
hsa05332:Graft-versus-host disease	7	7.97E−07	2.66E−06
hsa05330:Allograft rejection	7	1.62E−06	4.88E−06
hsa04630:Jak-STAT signaling pathway	10	1.40E−05	3.55E−05
22.CytokinE−chemokine_CNS	7	3.54E−05	0.001257
hsa04672:Intestinal immune network for IgA production	6	1.04E−04	2.18E−04

### Evaluation of our candidate essential proteins associated with COVID-19 pathology

The results of two previous subsections show that $Cut_1$ and $Cut_2$ sets are good candidates to find appropriate subsets that are related to COVID-19 pathology. In this subsection, two of these possible candidate subsets are evaluated. COVID-19 is a pandemic disease with a wide range of symptoms among different patients. What is clear is that the disease varies from asymptomatic to fatal in individuals. Recent studies show that the disease is more severe in people with underlying conditions such as Cardiovascular diseases, Diabetes, Hepatitis, Lung diseases, Kidney disease, and different types of cancers. Therefore, we expect that the underlying genetics of these diseases are associated with essential proteins that are associated with COVID-19. To find these essential proteins, we use gene-disease relation from Database for Annotation, Visualization, and Integrated Discovery (DAVID). Some proteins that are annotated to four out of five of these specific comorbid diseases in the $Cut_1$ and $Cut_2$ sets with significant $p-value$ are chosen as a set of essential proteins associated with COVID-19 ($E_1$ and $E_2$). Table 6 shows 76 and 79 essential proteins with the pathology of these comorbid diseases, respectively. In Fig. 11, we compare our candidate $E_1$ and $E_2$ sets with set of genes proposed by Dolan et al.^20^. This figure shows that 58 essential genes are approved by $E_1$, $E_2$ sets and also a set of genes proposed by Dolan et al. as essential proteins associated with COVID-19. We also evaluate the functional annotation by the performance of enrichment analysis on our candidate $E_1$ and $E_2$ sets. In Tables 7 and 8 the top significantly enrichment pathways for $E_1$ and $E_2$ sets are identified by DAVID analysis are reported, respectively. Finally, 93 proteins contain $E_1 \cup E_2$ are introduced as a final essential proteins set associated with COVID-19 disease pathology (See Table 6).

## Discussion and summary

COVID-19 pandemic, which is caused by acute respiratory syndrome (SARS-CoV-2), is currently causing irreparable harm to human life, so the world needs to quickly identify effective drugs to restrict the spread of the disease. One of the best ways to identify effective drugs in different diseases is to find proteins that are essential for the pathology of the diseases. The main idea of this paper is to find a set of proteins that are essential for the pathology of COVID-19 that can help us find some appropriate drugs. Therefore, in the first part of this work, we focused on finding the essential proteins of the virus targets or their associated modules in the human cells. For this purpose, we applied two algorithms to find the essential proteins associated with COVID-19 ($Cut_1$ and $Cut_2$). Both algorithms are based on finding the least number of proteins that are involved in the most biological processes associated with the virus and removing them causes the most disruption in the COVID-19 related biological networks. Then, we studied the set of proteins including the intersection and union of the results of these two algorithms and the intersection of each of these results with the targets of virus, as well as the set of virus targets and a set including the neighbors of the virus targets. Our results showed that out of 1373 biological processes related to COVID-19, 1306 biological processes have overlap with essential proteins in $S_2$ (Table 2). On the other hand, according to the definition of the set of biological processes related to COVID-19, the targets set of virus (*T*) with 332 proteins has overlap with all 1374 biological processes. Then, we need a more detailed analysis for the candidate sets of essential proteins. We evaluated the number of drugs used as an unapproved drug in COVID-19 (Covid-Drug) and targeted at least one of the proteins in these candidate sets. The results of our study showed that of among 17 drugs in the Covid-Drug group, 16 drugs target at least one of the proteins in $S_2$ set. From 17 drugs in this group, only *Ritonavir* and *Ibuprofen* target one of the proteins in *T* set which both of them are approved by $S_2$ set. As a result, the *T* candidate set cannot be a good candidate as the essential proteins sets compared to our proposed $S_2$ set. We also studied a group of drugs that are in the clinical trial phase (Clinical-Drug) and showed that 83$\%$ of them (273/328) target at least one protein of our candidate set. However, the *T* set confirms only 9$\%$ (30/328) of these drugs. Although the $Level_1$ set approves 86$\%$ (284/328) of these drugs, the low ratio of drugs to the number of proteins of this set shows that the $Level_1$ set cannot be a good candidate for essential proteins related to COVID-19 (Table 4). We also studied all of the drugs reported in UniProt that target at least one of the proteins in our PPI networks. The results of our study showed that our proposed $S_2$, $T_1$, $C_1$, $C_2$, $T_2$ and $S_1$ sets contain a significant percentage of drug targets (Table 5). The results of Tables  3, 4 and  5 show that out of 2,017 essential proteins obtained from Algorithm 1 ($Cut_1$) 22 drug targets are from the Covid-Drug group, 218 drug targets are from the Clinical-Drug group and 581 drug targets are from all All-Drug group. Also, among 2100 essential proteins obtained from Algorithm 2 ($Cut_2$), 20 drug targets are from the Covid-Drug group, 217 drug targets are from the Clinical-Drug group, and 539 drug targets are from the All-Drug group. In other words, the results of these two algorithms include a higher rate of target proteins than the $Level_1$ and *T* sets, considering the size of the sets. As a result, the outcomes of both algorithms can be identified as suitable candidate sets for COVID-19 related essential proteins sets. But, it is noticeable that not every essential protein is an appropriate candidate as an essential protein, because some of the essential proteins are related to the cellular function of the cell, and selecting them may lead to disruption of cellular functions. Therefore, we try to select candidate proteins that are essentials in disease pathology.

In the second part of this work, we focued on finding the essential proteins associated with COVID-19 pathology. To detect this set of essential proteins, we studied proteins that are associated with some underlying diseases. Since COVID-19 has more severe symptoms for patients with underlying diseases such as cardiovascular-related, hypertension, diabetes type 2, kidney-related diseases, and lung-related diseases. Identifying the proteins associated with these diseases that are in our essential proteins sets can be a suitable way to find essential proteins that are fundamentally related to COVID-19 pathology. Therefore, we selected the proteins presented in each of the candidate sets that are associated with at least four of the five underlying mentioned diseases. Our results showed that 76 essential proteins from the $Cut_1$ set and 79 essential proteins from the $Cut_2$ set are related to the mentioned diseases. These two sets are named $E_1$ and $E_2$, respectively. Finally, 93 proteins are introduced as essential proteins associated with COVID-19 disease pathology ($E_1 \cup E_2$). Our study showed that from these 93 proteins, only one protein, (**P09601**), was placed in the target set of virus (*T*) proteins and targeted by 15 drugs, including **NADH**. It is noticeable that this drug was not in the two groups of Covid-Drug and Clinical-Drug, but it has been approved in other studies recently^21^. Among these essential proteins, 7 proteins (**P01375, P08684, P10415, P10635, P15692, P35354, Q9NR96**) have been targeted by Covid-Drug group drugs. Out of 18 drugs in this group, 10 drugs including **Azithromycin, Ritonavir, Ibuprofen, Colchicine** and **Dexamethasone** were approved through these essential proteins. Besides, we found that 35 proteins out of 93 essential proteins were targeted by clinical drugs. We also found from 328 drugs in the Clinical-Drug group 185 drugs were approved by these 35 essential proteins, including **Baricitinib**^22^ and **Amlodipen**. Finally, we studied that for 65 out of 93 essential proteins associated with COVID-19 pathology, 1689 drugs including **Erythromycin** and **Letermorir** was introduced, which will be presented as future work. In addition, we analyzed the significant pathway enrichment for each of the candidate essential proteins sets. The results showed some signaling pathways enrichment related to COVID-19 (hsa04621; hsa04064; hsa04620); that have been introduced in the previous study^23^. There are also some significant disease-pathways (hsa05142; hsa05144; hsa05323; hsa05164; hsa05321) that have been presented in previous study^24^.

## Methods

### Dataset

In this work, we use five human high-throughput PPI network datasets. The first dataset, Huri, contains 52,548 human binary protein interactions^25^. The second one is gathered from the Biological General Repository for Interaction Datasets (BioGRID) and contains 296,046 interactions^26^. This dataset has several interactions that are obtained from different techniques. We only use the interactions that are represented as physical inactions and co-complexed proteins. The three other datasets are (Hippie^27^, Apid^28^, and Hint^29^) which contain 57,428, 17,1448 and 64,399 experimentally interactions, respectively. These interactions are derived from high-throughput yeast-two hybrid (Y2H) and mass spectrometry methods. All of the proteins from these five datasets are mapped to their corresponding Uniprot ID. If a protein could not be mapped to a Uniprot ID, it is removed. The final interactome that we used in this study contains 25,260 proteins and 30,4730 interactions. For each of these proteins, we use biological process terms from GO^17^ to point out the biological modules in human. We find that only 20,642 proteins from 25,260 or 81% of them are annotated. We use the IBP concept to avoid biases in the annotations that would potentially lead to incorrect conclusions. We consider a biological process annotation informative if it has the following two properties. First, it needs to have at least *k* proteins annotated with it. Second, each of its descendants GO terms needs to have less than *k* proteins annotated with them. In this study, we set three as a value of *k*. This yields to 1374 IBP GO terms related to 332 human proteins which are also identified as high-confidence SARS-CoV-2 Human PPI detected by Gordon et al.^3^ We also use all drugs and their targets reported in the UniProt website https://www.uniprot.org^30^ to evaluate our candidate sets as drug targets. This dataset contains 3064 proteins targets in our network. In addition, we use two groups of drugs related to COVID-19 reported in https://www.drugbank.ca website^19^.

### Algorithms for finding the essential proteins

Essential proteins perform a broad range of important functions in the biological network. Therefore, removing the minimum number of these essential proteins can have the highest impact on disrupting the biological activity of cells^31^. We proposed two different algorithms in previous works^15, 16^ for identifying the mentioned essential proteins. In this work, we modify the previous algorithms to find the essential proteins in the network that was created from a set of virus targets (332 proteins reported as possible targets of the SARS-CoV-2 virus^3^) and a set of processes associated with these proteins. For this purpose, we use biological information to build our network. Previous studies show that SARS-CoV-2 infects the human cells by generating 29 viral proteins that bind to different human proteins. Gorden et al.^3^ reveal 26 proteins from these 29 proteins and used affinity purification with the help of mass spectrometry leading to the identification of 332 human proteins involved in these viral proteins binds.

In the following section, we describe the details of algorithms 1 and 2. We explain the different parts of each algorithm in two separate sections. In both algorithms, the construction of the biological network is the same and is as follows: a biological network is considered as a weighted undirected graph $G= (V, E, \omega )$, where each node $v_i \in V$ represents a protein. Two proteins $v_i$ and $v_j$ are connected with an edge $e_{ij} \in E$ if they participate in the same biological process. The $\omega (e_{ij} )$ represents the weight of $e_{ij}$ which illustrates the number of biological processes that two proteins $v_i$ and $v_j$ participate in it. The degree of node $v_i$ shows the number of edges incident to this node.

#### Algorithm 1: betweenness value

In this algorithm, we try to impose maximum disruption to the network by selecting the least number of essential proteins with respect to the value called betweenness. For this purpose, we define the path and betweenness in the following. A path between two nodes in the graph is a sequence of edges that connect the number of distinct nodes through this path. In the weighted graph, the weight of the path is obtained from the sum of the weight of edges in this path, and the shortest path between two nodes is defined as a path with minimum weight. Having considered that, we can define the betweenness value for each node, $v_i$ , in the following way:

1 $$\begin{aligned} Bet\,w ({ v_i}) = \sum _{ { {v_j}, {v_k}} \in {V} } \frac{\chi _{e_{jk}} {v_i} }{\chi _{e_{jk}}} \end{aligned}$$

where $\chi _{e_{jk}}$ is the total number of shortest paths from node $v_j$ to node $v_k$ and $\chi _{e_{jk}} {v_i}$ is the number of shortest paths that pass through $v_i$. Algorithm 1 consists of three phases. In the first phase, the weighted graph is constructed as mentioned earlier. Then, the betweenness value for each node in the network is calculated. In the second phase, the input graph is partitioned into two disjointed parts. For this purpose, the node with minimum betweenness value is chosen as a candidate to put into a partition. Then, from all of the neighbors of this node, the node with the least betweenness value is selected and placed in the other partition, respectively. We continue this procedure recursively until all nodes are put into two nearly equal size partitions. In the third phase, we should select the minimum number of nodes for which their removal would destroy all crossing the edges between the two partitions. For this purpose, we select the nodes that are connected as the endpoints of the crossing edges between two parts with respect to their betweenness value. This phase is continued until the connected network is broken apart into two disjointed partitions. The third phase is equivalent to the minimum bi-section problem, which is a *NP*-complete problem^32^.

#### Algorithm 2: spectral partitioning

The Problem of partitioning a simple graph *G* into two balance or nearly balance partitions while minimizing the number of edges between these two parts (cut edge) is known as the *NP*-complete problem^33^ . Therefore, we approximate this balanced partitioning with the spectral bi-partitioning algorithm. This algorithm is based on eigenvectors of Laplace of the graph and divides the graph by two with respect to eigenvectors of a Laplacian matrix. Spectral partitioning is one of the most successful heuristic approaches in graph partition algorithms. Let $A= [a_{ij}]$ shows the adjacency matrix of *G* such that,

2 $$\begin{aligned} a_{ij} = {\left\{ \begin{array}{ll} 1 &{} \quad \textit{if } (v_i , v_j) \in { E} \\ 0 &{} \quad \textit{ otherwise} \end{array}\right. } \end{aligned},$$

We define a matrix $D = diag(d_i)$ as a diagonal degree matrix of *G*, in this matrix a $d(v_i)$ shows the degree of $v_i$ which is the number of edges incident to node $v_i$. Now, we consider the Laplacian matrix of the graph *G* by $L = D \backslash A$ and $L(G)=[l_{ij}]$ where,

3 $$\begin{aligned} l_{ij} = {\left\{ \begin{array}{ll} 1 &{} \quad \textit{if } (v_i , v_j) \in { E}\\ d(v_{i}) &{} \quad \textit{if } i=j\\ 0 &{} \quad \textit{ otherwise} \end{array}\right. } \end{aligned}.$$

The Laplacian matrix is a symmetric positive semi-definite matrix with some important properties. Let $u=(u_1, u_2, ..., u_n)$ be the normalized eigenvectors of matrix *L*(*G*) and $(\lambda _1, \lambda _2, \ldots , \lambda _n)$ be the corresponding eigenvalues of these eigenvectors. Then, the *u* is that pairwise orthogonal. If the graph *G* is a connected one, then $\lambda = \lambda _1$ is the only zero eigenvalue of L^33^.

Here we compute the eigenvectors of Laplacian matrix *L*(*G*), according to the second smallest eigenvalue of this matrix $\lambda _2$ and put them in vector $X=(x_1,\ldots , x_n)$. Next, we sort the elements of *X* and insert half of the nodes according to these elements in partition $G_1$ and the reminder of nodes in another partition $G_2$. The edges which cross these two partitions are the edge cut of our proposed graph *G*. The above procedure divides the nodes of graph *G* into two partitions $G_1$ and $G_2$ with nearly equal sizes and a set of cut edges $E(G_1, G_2)$ which connects these two partitions. We should disconnect these two partitions through these cut edges.

The remainder of the algorithm finding the vertex cut or a subset *C* from set *V* which has the following properties: (1) the set *C* is as small as possible; (2) the removal of *C* partitions graph *G* into two partitions $G_1 \backslash C$ and $G_2 \backslash C$ such that the ratio $|G_1 \backslash C |/| G_2 \backslash C |$, which shows the difference of the size of two subgraphs is as close to 1 as possible; and (3) for each cut edge $e_{ij} \in E(G_1 \backslash C,G_2 \backslash C)$, $v_{i} \in G_1 \backslash C$ and $v_{j} \in G_2 \backslash C$ . To find the vertex cut set *C*, suppose $M=\{ e_1= \alpha _1 \beta _1,\ldots ,e_m=\alpha _m \beta _m \}$ is the set of edges in cut edge $E(G_1, G_2)$ that found by the above mentioned algorithm. We construct the bipartite graph *H* that containing two partitions $G_1 \backslash C$ and $G_2 \backslash C$, with above procedure. Also, we consider each of the cut edges between two partitions $G_1$ and $G_2$ are having one endpoint in each part. For example, suppose that the *A* $= \{ \alpha _1,\ldots , \alpha _m \}$ is placed in $G_1$ and $B= \{ \beta _1,\ldots , \beta _m \}$ is placed in $G_2$, respectively. Then, we choose vertices from *A* and *B* with respect to their degrees repeatedly until the all the edges in *C* are removed.

## Data Availability

Datasets and the codes of the algorithms are available in our github repository (https://github.com/rosaaghdam/Drug-Target).
